# Efficacy of the Digital Therapeutic Mobile App BioBase to Reduce Stress and Improve Mental Well-Being Among University Students: Randomized Controlled Trial

**DOI:** 10.2196/17767

**Published:** 2020-04-06

**Authors:** Sonia Ponzo, Davide Morelli, Jamie M Kawadler, Nicola Rose Hemmings, Geoffrey Bird, David Plans

**Affiliations:** 1 BioBeats Group Ltd London United Kingdom; 2 Department of Engineering Science Institute of Biomedical Engineering University of Oxford Oxford United Kingdom; 3 Department of Experimental Psychology University of Oxford Oxford United Kingdom; 4 Social, Genetic and Developmental Psychiatry Centre Institute of Psychiatry, Psychology and Neuroscience King’s College London London United Kingdom; 5 Initiative in the Digital Economy Department of Science, Innovation, Technology, and Entrepreneurship University of Exeter Exeter United Kingdom

**Keywords:** anxiety, depression, mobile apps, biofeedback, mental health, mobile phones, technology

## Abstract

**Background:**

University students in the United Kingdom are experiencing increasing levels of anxiety. A program designed to increase awareness of one’s present levels of well-being and suggest personalized health behaviors may reduce anxiety and improve mental well-being in students. The efficacy of a digital version of such a program, providing biofeedback and therapeutic content based on personalized well-being metrics, is reported here.

**Objective:**

The aim of this study was to test the efficacy and sustained effects of using a mobile app (BioBase) and paired wearable device (BioBeam), compared with a waitlist control group, on anxiety and well-being in university students with elevated levels of anxiety and stress.

**Methods:**

The study employed a randomized, waitlist-controlled trial with assessments at baseline, 2 weeks, postintervention (4 weeks), and follow-up (6 weeks). Participants were eligible if they were current full-time undergraduate students and (1) at least 18 years of age, (2) scored >14 points on the Depression, Anxiety, and Stress Scale-21 items (DASS-21) stress subscale or >7 points on the DASS-21 anxiety subscale, (3) owned an iOS mobile phone, (4) did not have any previous psychiatric or neurological conditions, (6) were not pregnant at the time of testing, and (7) were able to read and understand English. Participants were encouraged to use BioBase daily and complete at least one course of therapeutic content. A *P* value ≤.05 was considered statistically significant.

**Results:**

We found that a 4-week intervention with the BioBase program significantly reduced anxiety and increased perceived well-being, with sustained effects at a 2-week follow-up. Furthermore, a significant reduction in depression levels was found following the 4-week usage of BioBase.

**Conclusions:**

This study shows the efficacy of a biofeedback digital intervention in reducing self-reported anxiety and increasing perceived well-being in UK university students. Results suggest that digital mental health interventions could constitute a novel approach to treat stress and anxiety in students, which could be combined or integrated with existing therapeutic pathways.

**Trial Registration:**

Open Science Framework (OSF.io) 2zd45; https://osf.io/2zd45/

## Introduction

### Background

Stress and anxiety in university students of the United Kingdom have been steadily rising in the past decade [1]. Research demonstrates that by the midpoint of their course, 9% of previously symptom-free students develop depression and 20% become anxious to clinically significant levels [2]. Nearly half (48%) of the students registered at a UK university-based general practice report high levels of anxiety [3]. Internationally, levels of students’ anxiety are also increasing, with university counseling services experiencing increasingly higher demand since 2010 [4-7]. Longitudinal studies report that students experience higher stress on entering university, which continues to increase during their studies, and does not return to previous levels after graduation [8,9].

Strikingly, only 25% to 36% of students with mental health issues seek treatment [10-12], largely due to the perceived stigma associated with these conditions [11,13]. A study investigating self-reported barriers to help-seeking behaviors and engagement in therapeutic pathways in students at risk of suicide found that a lack of time and a preference for self-management were among the main factors contributing to students’ choice not to seek treatment [14]. Untreated mental health issues among university students have been shown to have immediate and significant repercussions on the overall quality of life, increasing the likelihood of dropping out of university and committing suicide [1]. Importantly, untreated mental health issues during university years also have negative impact following graduation, affecting relationships, levels of productivity, and the likelihood of substance abuse [15].

Although on-site facilities are crucial for managing students’ mental health, their underutilization [16] suggests that novel approaches are needed to overcome accessibility barriers. Studies calling for more timely and preventative therapeutic interventions have highlighted the need for digital interventions [17-19]. The use of digital interventions, such as internet-based self-help resources and mobile apps, have been on the rise in the past decade due to their increased accessibility, availability, and anonymity [20-24], as well as their cost-effectiveness [25]. Owing to the widespread use of mobile phones, mobile apps could constitute effective therapeutic support for periods when students are away from the university, as well as increasing the capacity of on-site counseling services [26,27]. Mobile apps, paired with biosensors and wearable devices, are also effective in gathering passive data (eg, physical activity [28]) and self-report measures (eg, mood journaling). Accordingly, apps are increasingly used as a real-time monitoring tool, with personalized feedback, insights, and therapeutic content offered to users within the context of mental health interventions [29] and illness prevention [30].

A number of these digital interventions have proven effective in treating a variety of mental health disorders, ranging from anxiety and depression, to substance use disorder [31]. For example, an intervention lasting 2 weeks comprising brief, daily conversations and mood tracking with a Cognitive Behavioral Therapy (CBT)-oriented conversational agent (Woebot) found that, in comparison with an information-based digital control group, those in the Woebot group significantly reduced their symptoms of depression, while participants in both groups showed significantly reduced levels of anxiety [32]. Furthermore, an 8-week intervention in US university students with the mobile-app Calm was found to produce a significantly greater degree of stress reduction than that seen in a waitlist control group [23]. Despite these promising results, studies investigating the efficacy of a combined intervention, including both passive data collection and active therapeutic content, are still lacking.

The app BioBase (BioBeats, Ltd) aims at increasing individuals’ well-being by combining elements of mindfulness, biofeedback interventions (such as diaphragmatic breathing exercises), CBT, and behavioral activation theory [33-35]. Specifically, its psychoeducational content is based on the job demands-resources model, which has been shown to be associated with students’ well-being and stress management [36]. Alongside therapeutic content, data on physical activity, sleep quality, and heart rate are collected via a wrist-worn wearable device (BioBeam) and made available to individuals using the app to foster an increased awareness of users’ current well-being. Furthermore, available in-app tools include an ecological momentary assessment tool based on the Circumplex Model of Affect [37], allowing individuals to log their mood in the moment, and reflect back on their entries at a later date to gain insights into longer-term patterns of emotion. The app also includes diaphragmatic breathing exercises and relaxation techniques for in-the-moment stress reduction. In an initial feasibility study conducted with the BioBase app (BioBeats Ltd) in a sample of full-time employees [38], it was found that 4 weeks of usage of BioBase significantly reduced anxiety and increased self-reported mental well-being. The study also found that higher levels of baseline stress were associated with greater reductions in anxiety and increases in mental well-being, suggesting that usage of BioBase could be most beneficial for individuals with increased anxiety. However, the lack of a control group and the specificity of the selected population did not allow us to draw more general conclusions about the effects of using BioBase on self-reported anxiety and stress.

### Objectives

Hence, the purpose of this study was to test the efficacy of a 4-week intervention delivered via a mobile app and wearable device (ie, the BioBase program) in comparison with a waitlist control group on anxiety and general mental well-being in university students with elevated anxiety or stress. The study also examined sustained effects (at 6 weeks from baseline) of the intervention on anxiety and well-being. Finally, in the current study, measures of depression were collected to investigate the impact of the BioBase program on depressive symptoms.

We hypothesized that university students in the intervention group, but not in the waitlist control, would have significant improvements in anxiety and well-being following a 4-week intervention with BioBase. We also predicted that anxiety and well-being would have sustained effects in the intervention group, but not in the waitlist control, at 2 weeks following the end of the intervention. Furthermore, it was hypothesized that being enrolled in the BioBase program would reduce depressive symptoms after 4 weeks of usage.

## Methods

### Ethics Approval

This study was approved by an Institutional Ethics Committee at the University of Exeter (UEBS Research Ethics Committee, ethics application number: eUEBS002252). All participants provided informed, electronic consent prior to their enrollment in the study. Data from this study, including the preregistration protocol, are available on the Open Science Framework website (see Trial Registration section).

### Study Design

The current study was a randomized, waitlist control trial with assessments conducted at baseline, 2 weeks, postintervention (4 weeks), and follow-up (6 weeks). Participants randomly assigned to the intervention group took part in a 4-week well-being intervention (the BioBase program). Those assigned to the waitlist control group received the intervention after 8 weeks.

### Recruitment

Participants were recruited using institutional participant pools at different UK universities as well as via social media, mailing lists, and flyers and through university staff. Recruitment took place between October and November 2019, and potential participants were screened for eligibility via a Qualtrics survey. Inclusion criteria comprised being a full-time university student attending a university in the United Kingdom and (1) being aged between 18 and 25 years, (2) having scored >14 points on the Depression, Anxiety and Stress Scale-21 items (DASS-21 [39]) stress subscale or >7 points on the DASS-21 anxiety subscale, (3) owning an iPhone 6 or above, (4) not having any previous psychiatric or neurological conditions, (5) not being pregnant at the time of testing, and (6) being able to read and understand English. Participants were also excluded if they were currently in therapy or were using counseling services. Individuals taking part in the initial screening survey were entered into a lottery to win a UK £50 (US $63.93) Amazon Voucher.

### Randomization and Blinding

The original design was devised as a single-blind study; however, due to logistical reasons (ie, clarity of communications between the research team and participants) it was decided to unblind the design.

Eligible participants (n=262) were sent a reminder email prompting them to confirm their willingness to take part in the study. A total of 130 participants were randomly assigned to the intervention group and 132 participants were randomly assigned to the waitlist control group based on minimization factors: gender (2 categories: male and female), age (7 categories: 18, 19, 20, 21, 22, 23, 24, and 25 years), DASS-21 anxiety subscale (5 categories: normal, mild, moderate, severe, extremely severe), and DASS-21 stress subscale (5 categories: normal, mild, moderate, severe, extremely severe). DASS-21 categories were used for inclusion (ie, participants scoring within the normal range at screening were excluded from the study and those scoring normal at baseline were excluded from the analysis) and minimization purposes only. The first participant was allocated at random. Each subsequent participant’s group membership was allocated such that their addition to that group would lead to a closer match between the groups according to the minimization factors at screening. The random number list used to create the 2 groups was generated using the R *Minirand* package. Following randomization, the intervention group received their BioBeams (which are not functional until paired with a registered BioBase account) via post at their selected address.

#### Intervention Group

**Figure 1:**
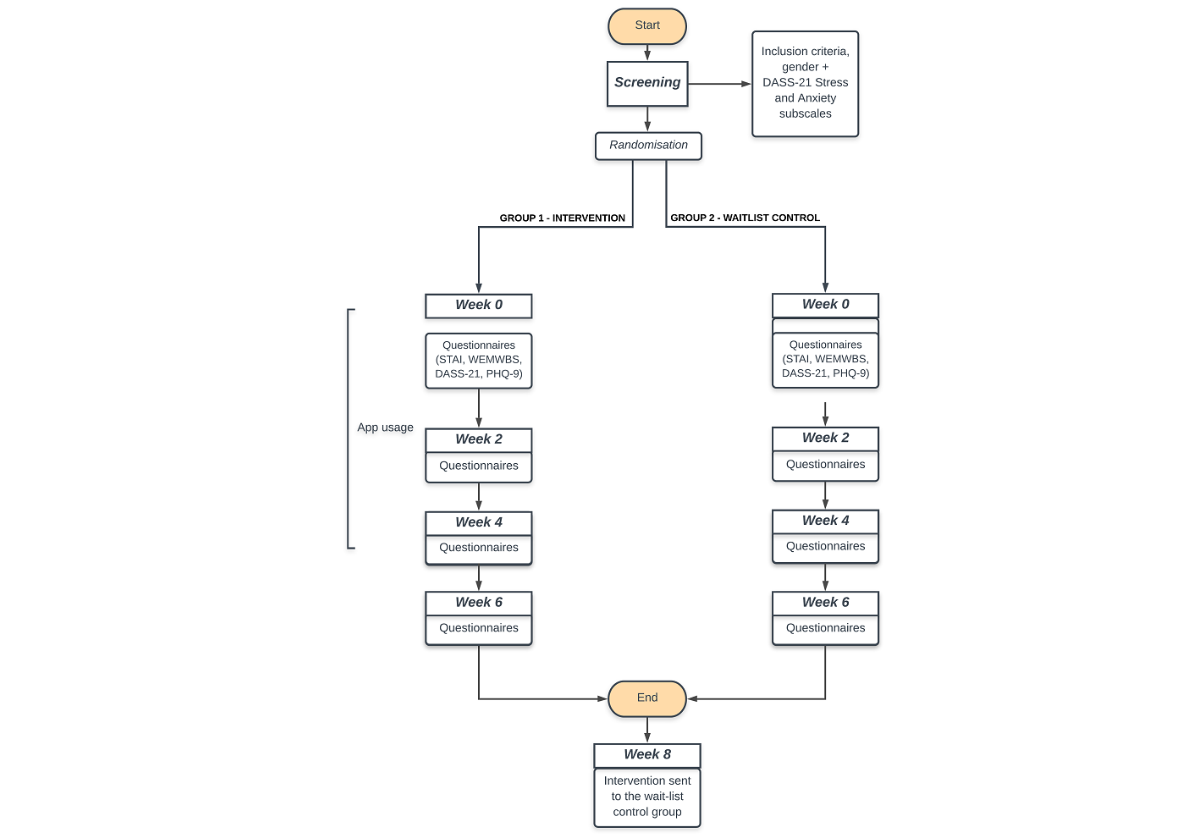
Timeline of the study. Participants were screened for inclusion and exclusion criteria. Of the 805 participants screened, only 262 were eligible to take part. These selected participants were subsequently randomly assigned to one of two groups: intervention or wait-list control. Each group received the questionnaires at baseline, 2, 4 and 6 weeks. The intervention group received access to the program following the first questionnaire completion.

After randomization, participants in the intervention group were emailed the first set of questionnaires to complete (Figure 1). At the end of the questionnaires, they were given details on how to download and register on the BioBase app.

The BioBase program is a multidimensional mobile app comprising psychoeducational content on mental health and well-being, mood tracking (via an ecological momentary assessment, EMA [40]), and in-the-moment exercises (eg, deep breathing and relaxation techniques). Furthermore, passive data on sleep, heart rate, and physical activity are collected via a wearable device (BioBeam) and presented to the users via a dashboard view.

The psychoeducational content is delivered via 42 five-min long modules, each tackling different aspects of psychological and emotional distress (see Multimedia Appendix 1 for a detailed description of the modules). The content is organized in three different courses, based on the job demands-resources model [41]. Each course relates to a different aspect of the model (ie, demands, control, and support) and it comprises 14 modules. Demands and control are widely recognized as relevant workplace stressors [42,43], while social support has been shown to positively impact perceived well-being [44]. Embedded in these modules are elements of CBT and self-compassion (see Multimedia Appendix 1). Digitally delivered CBT interventions have been proven efficacious in reducing the levels of anxiety and depression [45] and similarly, self-compassion has been shown to predict symptom severity in anxious and depressed individuals [46]. By incorporating these therapeutic elements, the courses aim to foster an individual’s recognition of internal physiological and emotional processes as a trigger for stress and identify effective coping strategies (eg, setting achievable goals aligned with the individual’s personal values).

The EMA tool allows an individual to report their mood in the moment by choosing a mood from a list of options, each with different valence (positive or negative) and arousal (high or low). Furthermore, individuals can specify any ecological component surrounding the moment they chose to declare their mood (ie, where they were, whether they were alone or with somebody, and what activities they were engaged with). EMAs are a valuable mood-tracking tool in the context of digital interventions specifically aimed at reducing levels of anxiety and depression (see [47] for a review).

The deep-breathing tool is designed as a quick intervention aimed at reducing stress and increasing relaxation and consists of 10 guided deep diaphragmatic breaths. Respiration biofeedback has been shown to lead to a reduction of symptoms of depression and anxiety (see [48] for a review on results of biofeedback interventions). Similarly, the body scan has been devised as a standalone quick relaxation intervention due to its effectiveness in reducing anxiety and depression (for a detailed review, see [49]).

Finally, passive data collection on physical activity (ie, number of steps performed every 20 seconds), sleep duration and quality (via a triaxial accelerometer with a sample rate of 100 Hz), and heart rate (via a photoplethysmography sensors) was obtained via the BioBeam wearable. This information was made available to participants via an in-app dashboard. Increased sleep awareness and implementation of sleep hygiene techniques have been recognized as a mediating factor in anxiety [50], thus supporting the notion that insights into individuals’ sleeping patterns may prove beneficial in stress reduction. Furthermore, as physical inactivity is associated with greater levels of anxiety [51], awareness of and insight into one’s own activity patterns may foster improvements in individuals’ well-being.

Participants were not prompted to use the app in any specific fashion and were left to freely engage with it for the whole intervention (ie, 4 weeks). Participants were, however, encouraged to continuously wear the BioBeam and engage with the therapeutic content (modules and tools) on a daily basis for at least 5 min. App usage was discontinued after the 4-week intervention ended.

#### Waitlist Control Group

The waitlist control group received the baseline questionnaire at the same time as the intervention group, followed by an email stating that they would be provided with the app and the wearable device in 8 weeks. Throughout the 8 weeks during which the intervention group used BioBase, the waitlist control participants received the 2-, 4-, and 6-week questionnaires, preceded by a reminder email to complete them. After 8 weeks, participants received a BioBeam at their selected address as well as an email with instructions on how to download and register the app.

### Measures and Incentives

Both groups completed four surveys via an online platform (Qualtrics). The surveys consisted of the following questionnaires: the State-Trait Anxiety Inventory (STAI-S-6 [52]), the Warwick-Edinburgh Mental Well-Being Scale (WEMWBS [53]), the DASS-21**,** and the Patient Health Questionnaire (PHQ-9 [54]). The DASS-21 Stress and Anxiety Subscales were used as a screening tool for participants’ inclusion in the study, whereas the depression subscale, together with the PHQ-9, was used as an outcome measure for depression. Demographic characteristics of the sample were collected at baseline. At the end of the study, each participant received a monetary incentive of £40 (£10 per each completed set of questionnaires at T0, T1, T2**,** and T3) plus an additional £5 if they decided to send back the wearable device received as part of the intervention.

#### Primary Outcome

The primary outcomes of the study were responses on the STAI [52]. The STAI-S-6 is a short version of the 10-item state subscale of the STAI. It is a 6-item scale, measuring state anxiety, with responses ranging from 1 (*Not at all*) to 4 (*Very much*). Scaled scores are obtained by multiplying the summed responses to each item by 20 and subsequently dividing the score by 6 (range 20-80).

#### Secondary Outcome

The secondary outcome of the current study was the WEMWBS [53], measuring perceived well-being. WEMWBS is a 14-item scale assessing subjective well-being and psychological functioning. Scoring is obtained by summing each response, ranging from 1 (*None of the time*) to 5 (*All of the time*) (range 14-70). WEMWBS has been validated for use in the United Kingdom with those aged 16 and above [53].

#### Additional Measures

Anxiety and stress were further measured via the DASS-21 subscales to ensure participants were still reporting elevated levels of stress or anxiety at baseline as well as during the screening procedure. Moreover, depression levels were investigated via the DASS-21 Depression subscale and the PHQ-9 questionnaire, a widely employed clinical tool. Although DASS-21 focuses on 1-week periods, PHQ-9 instructs individuals to report changes in the previous 2 weeks. Given that the focus on longer periods may mitigate the effects of random fluctuations in mood, both measures were collected.

### Statistical Analysis

#### Power

A power analysis, based on a previous feasibility pilot study, was conducted to estimate the required sample size for the randomized controlled trial. Accounting for potential dropout, the estimated sample size was at least 200 participants (100 per group), providing .95 power to detect a large effect size of .96 with an alpha of .05 in a final sample of at least 55 participants per group.

#### Data Exclusion

Given that the inclusion criteria for the current study comprised indication of anxiety or stress (as indexed by DASS-21 Anxiety and Stress subscale scores), 15 participants from the intervention group and 16 from the waitlist control group who initially scored above the normal range at screening but who scored in the normal range at baseline (T0) were excluded from statistical analysis. Participants were further excluded from final analyses if they did not download or open the app during the 4-week intervention as well as if they did not complete all questionnaires.

#### Data Analysis

The current study employed a mixed design with a between-subjects variable (group) with 2 levels: intervention versus waitlist control and a within-subjects variable (time) with 4 different levels: baseline, 2 weeks, 4 weeks, and 6 weeks. Given the advantages of linear mixed models (LMMs) in dealing with lack of homogeneity of variance and incomplete data sets across time points [55], LMMs were used to analyze our primary and secondary outcomes. Specifically, group and time were the fixed effects and time/subjects were the nested random effects. Planned comparisons (paired-samples *t* tests) were conducted to explore the direction of significant interactions between group and time. Effect sizes for planned comparisons were calculated using Cohen *d* (pooled SD) to allow maximum comparability with previous research [56]. The *P* values reported later have not been corrected for multiple comparisons but remain significant if corrected. Data were analyzed and plotted using the *tydiverse*, *ggplot2* [57], *lmer4* [58], and *lmerTest* [59] packages for R.

## Results

### Participant Enrollment and Demographics

**Figure 2:**
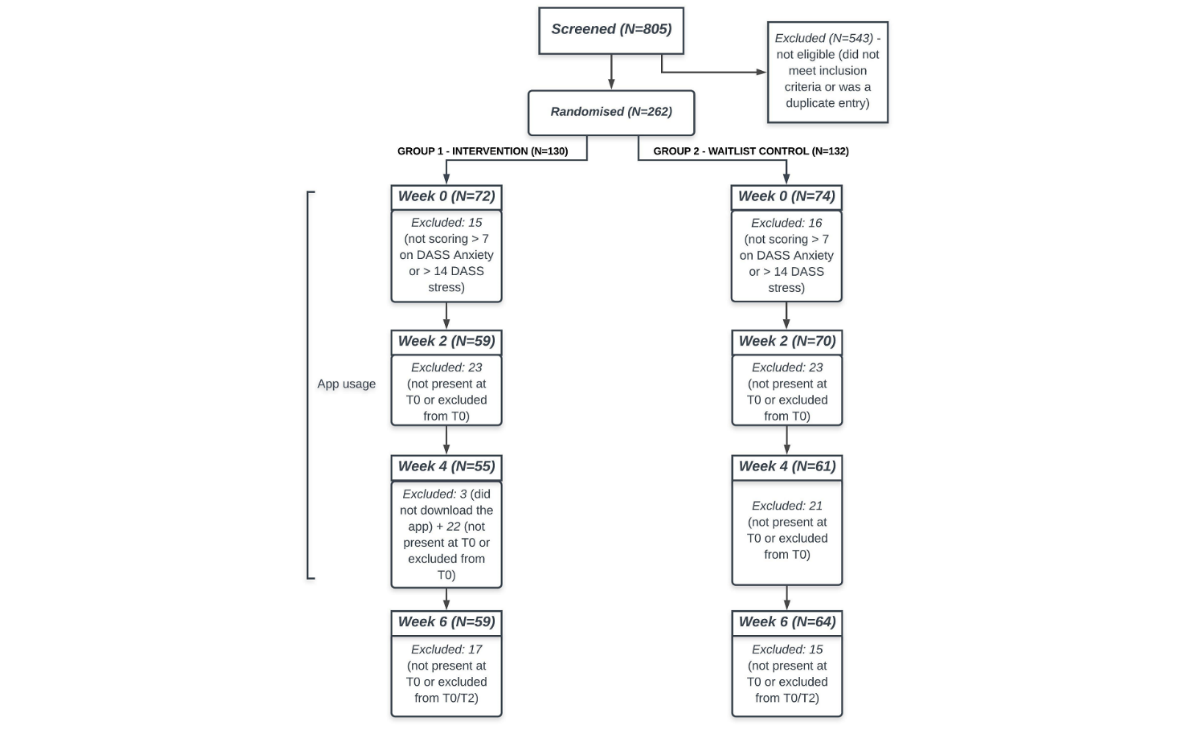
Flowchart of enrollment and retention rates throughout the study in the intervention and wait-list control groups.

**Figure 3:**
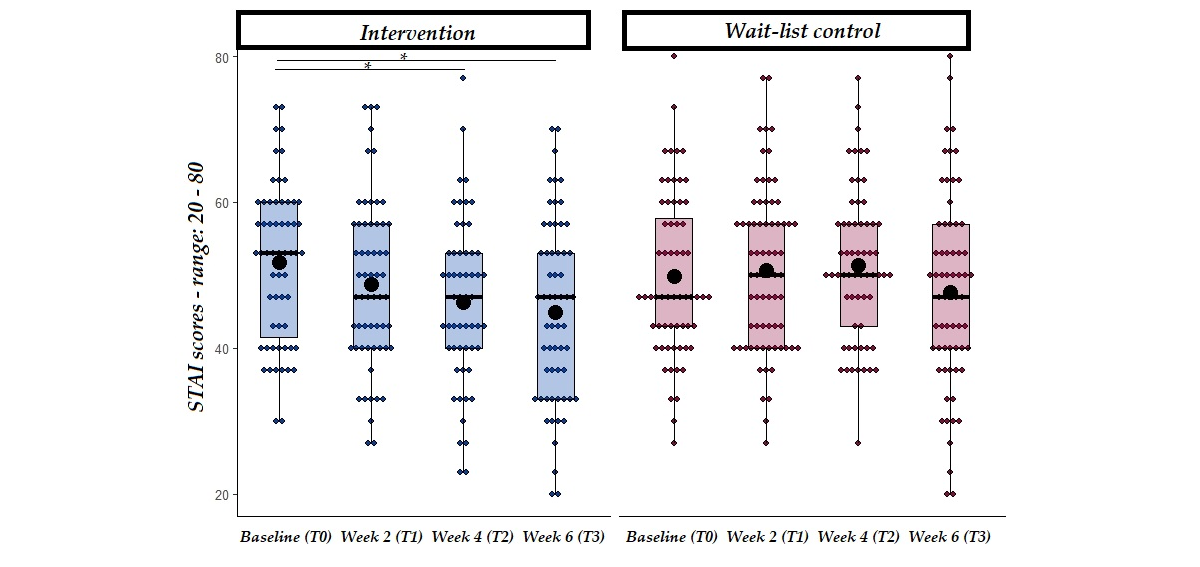
STAI scores at baseline, week 2, week 4 and week 6 follow-up from the start of the intervention in both intervention and wait-list control groups. Solid line=median; black dot=mean; whiskers: upper whisker=min(max(x), Q_3 + 1.5 x IQR); lower whisker=max(min(x), Q_1 - 1.5 x IQR). *P<.01.

**Figure 4:**
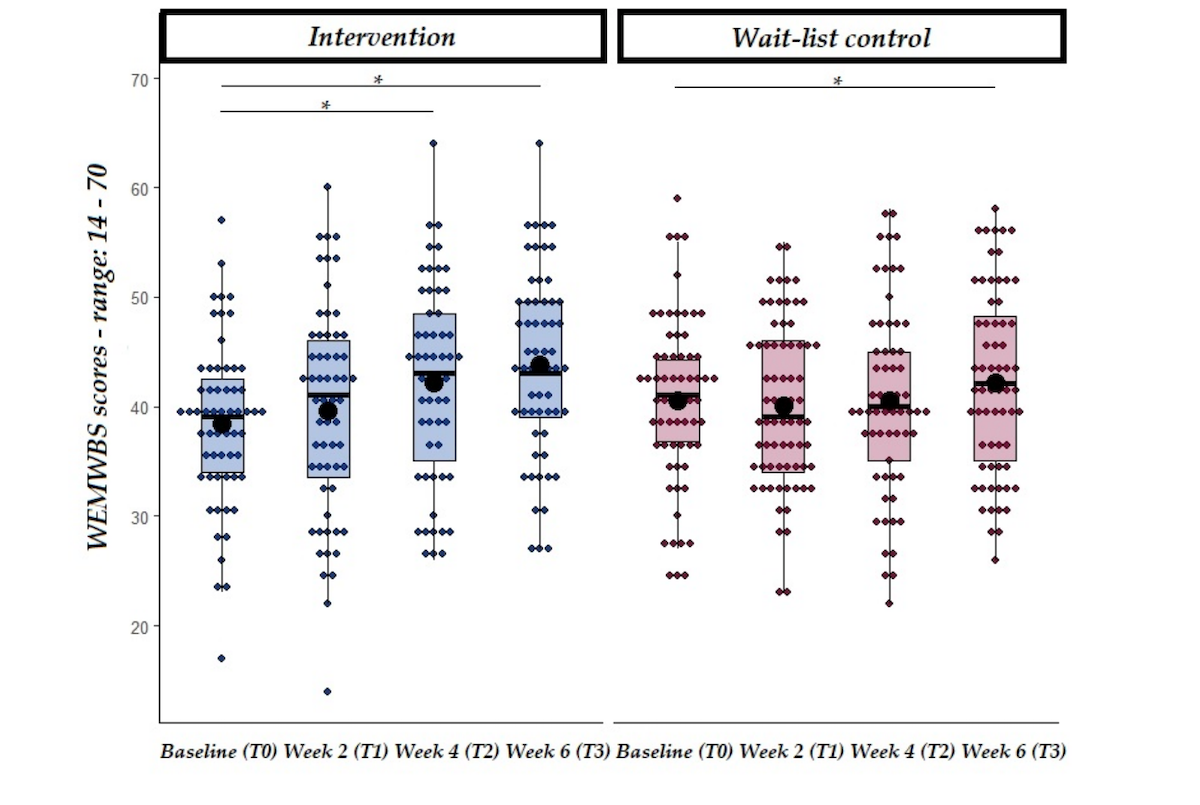
WEMWBS scores at baseline, week 2, week 4 and follow-up from the start of the intervention in both intervention and wait-list control groups. Solid line=median; black dot=mean; whiskers: upper whisker=min(max(x), Q_3 + 1.5 x IQR); lower whisker=max(min(x), Q_1 - 1.5 x IQR). *P<.05.

Figure 2 illustrates the flow of participants through the study and reasons for exclusion. Of 805 participants that were screened via an online questionnaire for inclusion and exclusion criteria, 262 participants were deemed eligible and were randomized into either the intervention (n=130) or waitlist control (n=132) groups. Of those, 59 participants from the intervention group and 64 from the control group completed the final questionnaire at T3 and were included in the analysis. Engagement data from the participants in the intervention group showed participants engaged with the app 21.9 of 29 days on average (median 26 days, IQR 13 days, range: 2-29 days). On average, participants engaged with the app 5.33 (SD=5.03) minutes per day (range: 2.13-28.68) over the 29 days of the intervention (see Figures 1 and 2, Multimedia Appendix 2). However, no correlation was found between the total amount of engagement with the app and differences, from baseline to T2 (4-weeks), in the main outcome measures (see Figures 3 and 4, Multimedia Appendix 3).

Participants in the two groups did not differ significantly with respect to age, gender, nor their levels of stress and anxiety at baseline (see Table 1). A total of 59 participants from the intervention group and 70 from the control group partook in the second questionnaire (intervention group: 38 females, age range: 18-25 years, mean 19.9 years, SD 1.9; waitlist control: 48 females, age range: 18-25 years, mean 19.9 years, SD 1.89; Figure 2), 55 and 61 (respectively) in the third (intervention group: 36 females, age range: 18-25 years, mean 19.9, SD 1.82; waitlist control: 43 females, age range: 18-25 years, mean 19.93 years, SD 1.95); and finally 59 and 64 participants completed the follow-up questionnaire (intervention group: 38 females, age range: 18-25 years, mean 19.92 years, SD 1.86; waitlist control: 43 females, age range: 18-25 years, mean 20, SD 1.90).

**Table 1:** Summary of participant characteristics at baseline (T0).

Characteristics		Intervention group (n=72)	Waitlist control (n=74)	*P* value
**Gender (n)**				
	Females	45	47	N/A^a^
	Males	27	27	N/A
Age (years), mean (SD)		19.9 (1.83)	19.84 (1.76)	.83
DASS-21^b^ anxiety, mean (SD)		15.39 (6.68)	14.46 (7.23)	.42
DASS-21 stress, mean (SD)		21.08 (7.02)	19.86 (7.66)	.32

^a^N/A: not applicable.

^b^DASS-21: Depression, Anxiety, and Stress Scale-21 items.

### Primary Outcomes

#### State-Trait Anxiety Inventory: Baseline to Follow-Up

The primary hypothesis was that, in comparison with the waitlist control group, the intervention group treated with BioBase would show a significant reduction in anxiety levels (measured via STAI-S-6) at the end of the intervention (ie, 4 weeks following baseline measures). Furthermore, it was hypothesized that such effects would be sustained at follow-up (ie, 2 weeks after the end of the intervention). An LMM with STAI-S-6 as the dependent variable and group and time (as well as their interaction) as independent variables was carried out. This analysis revealed a significant main effect of time (at both week 4 and week 6), with scores being lower in comparison with baseline. Furthermore, a significant interaction between group and time (at both week 4 and week 6) on perceived anxiety levels (see Table 2 for a summary of the LMMs) was observed.

To further explore the significant interaction between time and group, planned comparisons were conducted separately in the intervention and waitlist control groups comparing STAI-S-6 values at baseline with week 4 and follow-up (6 weeks), respectively. Findings revealed that STAI-S-6 at week 4 was significantly lower in the intervention group but not in the control group (see Table 3 for a summary of descriptive statistics and planned comparisons and Figure 3) and that such a reduction was still present at follow-up in the intervention group only.

One of our secondary hypotheses was that, in line with the results from Fitzpatrick and colleagues [32], the BioBase intervention would show efficacy in decreasing self-reported levels of anxiety after 2 weeks of treatment compared with the control group. However, no interaction between time and group was found at week 2, suggesting that changes in anxiety did not occur within the first 2 weeks of the intervention (Table 2).

**Table 2:** Summary of the linear-mixed model on State-Trait Anxiety Inventory scores over the 4 time points in the intervention and waitlist control groups.

Predictors		STAI-S-6^a^		
		Estimates	95% CI	*P* value
Intercept		53.61	47.07-60.15	<.001
Group		−1.90	−5.99 to 2.19	.36
T1—2 weeks		−6.35	−12.87 to 0.17	.06
T2—4 weeks		−12.25	−18.90 to −5.59	*<.001* ^b^
T3—6 weeks		−11.32	−17.84 to −4.81	*.001*
Group: Time T1		3.44	−0.65 to 7.52	.10
Group: Time T2		7.09	2.94 to 11.24	*.001*
Group: Time T3		4.56	0.49 to 8.63	*.03*
**Random effects**				
	*σ* ^2^	16.82	N/A^c^	N/A
	*τ*00 Time: Participants ID	49.44	N/A	N/A
	*τ*00 Participants ID	67.20	N/A	N/A
	ICC^d^	0.87	N/A	N/A
	N time	4	N/A	N/A
	N ID	123	N/A	N/A
	Observations	491	N/A	N/A
	Marginal *R*^2^/conditional *R*^2^	0.037/0.879	N/A	N/A

^a^STAI: State-Trait Anxiety Inventory.

^b^Italicized values are significant.

^c^N/A: not applicable.

^d^ICC: intraclass correlation coefficient.

**Table 3:** Mean, SD, and planned comparisons on State-Trait Anxiety Inventory scores over the duration of the study (T0, T1, T2, and T3) in the intervention and waitlist control groups.

Time point	STAI-S-6^a^: planned comparisons							
	Intervention group				Waitlist control			
	Mean (SD)	*t* test (*df*)	*P* value	Effect size, *d*	Mean (SD)	*t* test (*df*)	*P* value	Effect size, *d*
T0—baseline	51.71 (10.78)	N/A^b^	N/A	N/A	49.81 (10.96)	N/A	N/A	N/A
T1—week 2	N/A	N/A	N/A	N/A	N/A	N/A	N/A	N/A
T2—week 4	46.31 (11.32)	3.507 (54)	<.001	0.67	51.33 (10.35)	−1.449 (60)	.15	0.26
T3—week 6	44.95 (12.52)	4.35 (58)	<.001	0.81	47.61 (13.29)	1.542 (63)	.13	0.27

^a^STAI-S-6: State-Trait Anxiety Inventory.

^b^N/A: not applicable.

### Secondary Outcomes

#### Warwick-Edinburgh Mental Well-Being Scale: Baseline (T0)—Follow-Up (T3)

One of the secondary hypotheses was that participants in the intervention group only would report higher levels of well-being (as measured by WEMWBS) at both the end of the intervention and at follow-up. An LMM with WEMWBS as the dependent variable and group and time (as well as their interaction) as the independent variables revealed a significant main effect of time (at both week 4 and week 6), suggesting that perceived well-being increased over time regardless of groups. Furthermore, a significant interaction between group and time (week 4 and week 6) was found (see Table 4), which was further analyzed with planned comparisons. *t* tests were conducted separately in the intervention and waitlist control group comparing WEMWBS values at baseline (T0) and following the 4-week intervention as well as at follow-up (week 6). Results showed that in the intervention group only, WEMWBS values significantly increased between baseline and week 4, suggesting a higher perceived well-being in the intervention group (see Table 5 and Figure 4). WEMWBS values significantly increased between baseline and follow-up in both groups, but with higher values on average in the intervention group, suggesting an increase in perceived well-being.

**Table 4:** Summary of the linear-mixed model on Warwick-Edinburgh Mental Well-Being Scale scores over the 4 time points in the intervention and waitlist control groups.

Predictors			WEMWBS^a^				
			Estimates		95% CI		*P* value
Intercept			36.40		31.65 to 41.16		*<.001* ^b^
Group			2.07		−0.90 to 5.04		.17
T1—2 weeks			2.69		−1.25 to 6.64		.18
T2—4 weeks			7.41		3.38 to 11.44		*<.001*
T3—6 weeks			8.94		4.99 to 12.88		*<.001*
Group: Time T1			−1.57		−4.05 to 0.90		.21
Group: Time T2			−3.96		−6.47 to −1.45		*.002*
Group: Time T3			−3.65		−6.11 to −1.18		*.004*
**Random effects**							
	*σ* ^2^	5.00		N/A^c^		N/A	
	*τ*00 Time: Participants ID	19.25		N/A		N/A	
	*τ*00 Participants ID	46.28		N/A		N/A	
	ICC^d^	0.93		N/A		N/A	
	N time	4		N/A		N/A	
	N ID	123		N/A		N/A	
	Observations	533		N/A		N/A	
	Marginal *R*^2^/conditional *R*^2^	0.029/0.794		N/A		N/A	

^a^WEMWBS: Warwick-Edinburgh Mental Well-Being Scale.

^b^Values in italics are significant.

^c^N/A: not applicable.

^d^ICC: intraclass correlation coefficient.

**Table 5:** Mean, SD, and planned comparisons on Warwick-Edinburgh Mental Well-Being Scale scores over the duration of the study (T0, T1, T2, and T3) in the intervention and waitlist control groups.

Time point	WEMWBS^a^—planned comparisons							
	Intervention group				Waitlist control			
	Mean (SD)	*t* test (*df*)	*P* value	Effect size, *d*	Mean (SD)	*t* test (*df*)	*P* value	Effect size, *d*
T0—Baseline	38.47 (7.54)	N/A^b^	N/A	N/A	40.55 (7.76)	N/A	N/A	N/A
T1—week 2	N/A	N/A	N/A	N/A	N/A	N/A	N/A	N/A
T2—week 4	42.15 (9.02)	−3.385 (54)	.001	0.65	40.51 (8.64)	0.814 (62)	.42	0.15
T3—week 6	47.76 (8.31)	−6.260 (58)	<.001	1.16	42.19 (8.37)	−2.127 (63)	.04	0.38

^a^WEMWBS: Warwick-Edinburgh Mental Well-Being Scale.

^b^N/A: not applicable.

#### Additional Measures

**Figure 5:**
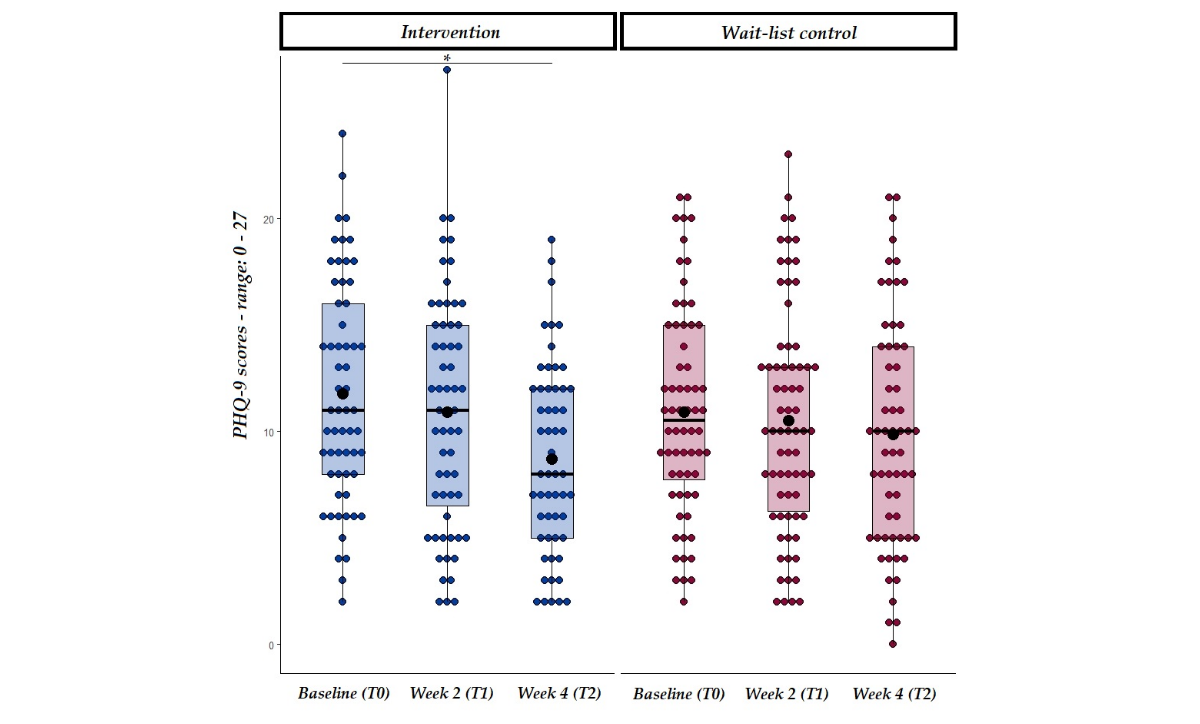
PHQ-9 scores at baseline and week 2 from the start of the intervention in both intervention and wait-list control groups. Solid line=median; black dot=mean; whiskers: upper whisker=min(max(x), Q_3 + 1.5 x IQR); lower whisker=max(min(x), Q_1 - 1.5 x IQR). *P<.01.

To explore the potential of the BioBase program to reduce depression over the 4-week period of use, depression was measured via the PHQ-9 questionnaire and a linear mixed model with depression scores as the dependent variable and group and time (as well as their interaction) as the independent variables was carried out. This analysis revealed that depressive symptoms decreased at 4-weeks from the start of the intervention, regardless of groups, but that in the intervention group this effect was more pronounced (as suggested by the significant interaction between group and time at week 4). This significant interaction (Table 6) was further analyzed via planned comparisons on depression scores in the intervention and waitlist control group at baseline and following the 4-weeks intervention. Findings revealed that in the intervention group only, PHQ-9 values significantly decreased between baseline and week 4, suggesting a lower perceived level of depression (see Table 7 and Figure 5). Changes in the Depression subscale of the DASS-21 were also explored. This analysis revealed a main effect of Time at both week 2 and week 4 (see Multimedia Appendix 4), with depression levels reducing over time irrespective of groups. Although the same pattern highlighted by the PHQ-9 scores was observed (intervention group: Baseline: mean 18.58, SD 10.87; week 4: mean 12.76, SD 8.77; waitlist control: baseline: mean 16.44, SD 9.67; week 4: mean 12.16, SD 8.90), there was no significant interaction between Group and Time. Such finding could be due to the intrinsic characteristics of the scales (ie, DASS-21 focuses on 1-week periods, while PHQ-9 asks individuals to report changes in the previous 2-weeks).

**Table 6:** Summary of the linear mixed model on Patient Health Questionnaire scores over the duration of the intervention (T0, T1, and T2) in the intervention and waitlist control groups.

Predictors		PHQ-9^a^		
		Estimates	95% CI	*P* value
Intercept		12.65	9.73-15.58	*<.001* ^b^
Group		−0.87	−2.70 to 0.95	.35
T 1—2 weeks		−1.34	−3.53 to 0.84	.23
T 2—4 weeks		−4.91	−7.15 to −2.67	*<.001*
Group: Time T1		0.46	−0.90 to 1.83	.51
Group: Time T2		2.07	0.67 to 3.46	*.004*
**Random effects**				
	*σ* ^2^	4.25	N/A^c^	N/A
	*τ*00 Time:Participants ID	3.22	N/A	N/A
	*τ*00 Participants ID	19.22	N/A	N/A
	ICC^d^	0.84	N/A	N/A
	N time	3	N/A	N/A
	N ID	123	N/A	N/A
	Observations	368	N/A	N/A
	Marginal *R*^2^/conditional *R*^2^	0.026/0.845	N/A	N/A

^a^PHQ-9: Patient Health Questionnaire.

^b^Value in italics are significant.

^c^N/A: not applicable.

^d^ICC: intraclass correlation coefficient.

**Table 7:** Mean, SD, and planned comparisons on Patient Health Questionnaire scores over the duration of the intervention (T0, T1, and T2) in the intervention and waitlist control groups.

Time point	PHQ-9^a^—planned comparisons							
	Intervention group				Waitlist control			
	Mean (SD)	*t* test (*df*)	*P* value	Effect size, *d*	Mean (SD)	*t* test (*df*)	*P* value	Effect size, *d*
T0—baseline	11.78 (5.2)	N/A^b^	N/A	N/A	10.91 (4.93)	N/A	N/A	N/A
T1—week 2	N/A	N/A	N/A	N/A	N/A	N/A	N/A	N/A
T2—week 4	8.71 (4.45)	5.139 (54)	<.001	0.99	9.85 (5.38)	1.392 (60)	.17	0.25

^a^PHQ-9: Patient Health Questionnaire.

^b^N/A: not applicable.

## Discussion

### Principal Findings

The aim of the current study was to investigate the efficacy of BioBase, a 4-week app-based intervention, in reducing anxiety and increasing well-being in university students with high self-reported levels of stress or anxiety. Results revealed that using the BioBase program for 4 weeks led to reduced self-reported anxiety and increased self-reported well-being. Such results were sustained at follow-up, with participants in the intervention group maintaining lower levels of self-reported anxiety and higher levels of well-being at 6 weeks from the study start date. Effect sizes ranged from moderate to large throughout the different outcomes.

### Comparison With Prior Work

The primary hypotheses of the current study were that in the intervention group only, levels of anxiety would decrease following enrollment in the BioBase program and that this reduction would be sustained after 2 weeks from the end of the intervention. In line with our first primary hypothesis, we found that self-reported levels of anxiety were significantly reduced in the intervention group after 4 weeks of app usage. This finding is in line with results from previous studies using digital interventions in both student [23] and nonstudent [32] populations. As mentioned in the Introduction section, Huberty et al [23] found that the mobile app Calm, consisting of a guided mindfulness meditation program, was effective in reducing stress levels among university students. In contrast to the BioBase 4-week program of 5 min a day; however, the Calm intervention was an 8-week program, requiring participants to first complete a 1-week course and then actively engage with the therapeutic content for at least 10 min a day. The efficacy of the BioBase program despite the reduced *dosage* may be related to the nature of the BioBase program: the therapeutic content is only one aspect of the hypothesized factors at play in anxiety reduction. Interactions with the app dashboard (showing participants their levels of activity, sleep quality, mood declarations over time, and heart rate), as well as usage of the tools, are hypothesized to be causally efficacious in addition to the traditional therapeutic content. Future studies using BioBase could shed light on the individual contribution of each of these aspects in reducing anxiety levels.

These results are also in line with previous findings [38], suggesting a significant reduction in anxiety following a 4-week intervention with the BioBase program in a sample of full-time employees. However, in this previous study, the effect of the intervention was not assessed beyond the end of the program. In the current study, we showed that the effect of the intervention persisted for 2 weeks following the end of the program. This result, in line with previous research [23], highlights the efficacy of mobile apps to reduce stress and anxiety over time, and their potential to supplement existing therapeutic support [18,27,29,30]. Future studies should investigate the extent to which these effects persist over longer timeframes, with the aim of identifying optimal guidelines for engagement to maximize outcomes.

A secondary hypothesis was that reduction in anxiety would be present following 2 weeks of enrollment in the BioBase program in the intervention group (but not in the waitlist control group). However, we did not find evidence of efficacy at 2 weeks. This finding is in contrast with a previous study conducted in the young adult population [32], using a CBT-based intervention to reduce anxiety and depression, which found significant results following 2-week long interactions with a Web-based conversational agent. Nevertheless, the current study significantly differed in both methods of delivery (app vs Web-based) as well as type of intervention. Although Fitzpatrick and colleagues employed a daily intervention, comprising specific time windows of interaction with the therapeutic content, the current study had a more ecological approach, with the BioBase program being available to participants at all times yet not being a daily commitment. Thus, the reason behind the lack of efficacy following a 2-week enrollment in the program may be due to differences in perceived benefit from the participants’ perspective, that is, it may be easier to recognize the impact of a daily conversational intervention versus a natural, progressive engagement with a multidimensional program. Further research, comparing different kinds of interventions, would be needed to shed light on these findings.

In terms of secondary outcomes, it was hypothesized that perceived well-being would increase following a 4-week intervention with BioBase and that this effect would be sustained at follow-up (6 weeks). As predicted, we found that participants in the intervention group reported higher levels of perceived well-being after 4 weeks, which were still significant at 2 weeks from the end of the intervention. Nevertheless, we also found a main effect of time, with levels of perceived well-being being higher at T2 and T3, regardless of the grouping. Further studies with single- or double-blind designs could investigate the impact of being enrolled in a study on perceived well-being.

Finally, additional measures of depression were obtained via the PHQ-9 questionnaire and DASS-21 Depression subscale to assess the feasibility of the BioBase program in reducing depressive symptoms. Results showed that participants taking part in the current study reported lower depression levels after 4 weeks of BioBase usage and sustained effects at follow-up (as measured via the PHQ-9). Nevertheless, despite showing the same pattern of reduction, the same results were not significant for the DASS-21 Depression subscale. Such a discrepancy may be due to differences in sensitivity of the 2 measures, given the focus on periods of different length, and further research is needed to shed light on these findings. Furthermore, given that the trial was conducted in November 2019 through December 2019, it is possible that the reduction in DASS Depression scores observed in the waitlist control group could be due to changes in university work demands, such as coursework deadlines and exams, over this period.

This result is nonetheless particularly relevant when assessing the lack of engagement of individuals at risk of suicide with established pathways of support. Specifically, the possibility to access a digital mental health intervention, which could be efficacious in reducing depressive symptomatology could, represent a novel approach in students at risk of suicide [1,14]. Future studies should specifically investigate the efficacy of such intervention in a student population with individuals suffering from self-reported depressive symptoms.

### Limitations

A limitation of the current study is the lack of a blinding procedure. As mentioned in the Methods section, the current study was an unblinded, randomized controlled trial, with participants in the control group being aware of the fact that they were not currently partaking in the intervention. This was a consequence of the type of control group employed. However, both groups received the same kind of communications and were prompted to respond to the questionnaires in the same way. A targeted standardi**z**ed email was sent every week, with the timeline of the study and key dates as a reminder to participants. Although these measures reduced the possibility that unblinding could influence the results of the current study, future studies should investigate the extent to which being enrolled in an intervention leads to improvements in anxiety and well-being by employing a single-blind design, with an information-based control group.

Moreover, owing to lack of data on ethnicity, or information on the characteristics of the students underusing mental health services, it was not possible to assess the generalizability of our sample. Further studies should further investigate this, by replicating the current study while controlling for these variables.

Furthermore, in the current study, it was not possible to differentiate the effect of the different components of the BioBase program. Although this is a characteristic of digital interventions [60], future studies should explore what components of the BioBase program are most efficacious for which individuals.

In addition, the current study targeted subclinical levels of anxiety; therefore, participants with a psychiatric diagnosis of anxiety were excluded. This decision was made to explore symptom reduction and well-being increase without the confounding factors of being currently in treatment for anxiety. It could be the case, however, that effect sizes were underestimated if BioBase is more efficacious in participants with higher anxiety levels. Further research is needed to better understand the potential effects of BioBase in individuals with a clinical diagnosis of anxiety or stress.

In terms of the follow-up measure, the current study employed a 6-weeks follow-up, aimed at investigating the sustained effects of the intervention. However, it should be noted that further research is needed to explore long-lasting effects of the intervention (eg, 8 weeks).

Finally, in the current study, no specific criterion was used with regard to app usage. Given that we wanted to observe how participants would naturally engage and interact with the program, there was no strict indication nor control on participants’ way to use the app. Nevertheless, the majority of the sample engaged with the intervention, with only 3 people not downloading or installing the app. Future research could explore whether a more controlled intervention, with specific engagement criteria, could lead to more efficacious results while still maintaining ecological validity.

### Conclusions

In this study, we showed that a 4-week digital intervention was efficacious in reducing anxiety and increasing well-being in a student population with high levels of self-reported stress and anxiety. These effects were sustained after 2 weeks from the end of the intervention, thus suggesting prolonged efficacy over time. To the best of our knowledge, this is the first study showing the efficacy of a multidimensional digital program, comprising therapeutic content, biofeedback, and mood-journaling, in reducing anxiety and increasing well-being in a student population. These findings are particularly relevant given the documented preference of students to self-help, rather than accessing on-site facilities, when facing mental health issues. Furthermore, the common use of mobile phones makes this type of intervention both accessible and scalable for higher education institutions who aim to extend the support provided to their students [27]. Future research should investigate the feasibility of including digital mental health interventions in the existing therapeutic pathways, thus encouraging preventative as well as intervention-driven approaches to mental health, tailored to the needs of the individuals.

## Appendix

Multimedia Appendix 1 Content and theoretical contextualization of the three psycho-educational courses contained in BioBase.

Multimedia Appendix 2 Summary of the Linear Mixed Model on DASS-21 Depression scores over the duration of the intervention (T0, T1 and T2) in the intervention and wait-list control groups.

Multimedia Appendix 3 App Engagement figures.

Multimedia Appendix 4 Engagement time and differences in anxiety and wellbeing.

Multimedia Appendix 5 CONSORT EHEALTH checklist (v 1.6.1).

## Abbreviations

CBT: cognitive behavioral therapy
DASS-21: Depression, Anxiety and Stress Scale-21 items
EMA: ecological momentary assessment
LMM: linear mixed model
PHQ: Patient Health Questionnaire
STAI: State-Trait Anxiety Inventory
WEMWBS: Warwick-Edinburgh Mental Well-Being Scale
