# Supplementary material for: Efficacy of the Digital Therapeutic Mobile App BioBase to Reduce Stress and Improve Mental Well-Being Among University Students: Randomized Controlled Trial
Source: JMIR Mhealth Uhealth. 2020 Apr 6;8(4):e17767. doi: 10.2196/17767 (PMC7171562; doi:10.2196/17767)
Supplement: Multimedia Appendix 1 [file mhealth_v8i4e17767_app1.docx]

Multimedia Appendix 1. Content and theoretical contextualization of the three psycho-educational courses contained in BioBase.

| **Course title and HSE/JDR content theme covered** | **Aims and objectives of each course** | **Course-targeted HSE questions** | **Modules covered** |
| --- | --- | --- | --- |
| Managing your Workload - Demands | AIMS: To reduce the perceived stress experienced due to overwhelming demands.  Objectives: To recognise stress is an imbalance of demands and resources. To recognise and categorise current demands and resources. To reduce demands and increase resources through time management techniques. | Different groups at work demand things from me that are hard to combine  I have unachievable deadlines  I have to work very intensively  I have to neglect some tasks because I have too much to do  I am unable to take sufficient breaks  I am pressured to work long hours  I have to work very fast  I have unrealistic time pressures | JDR model^1^; basic psychological needs (Mastery)^2^; time management techniques^3^; concept of ‘Flow’^4^ |
| Taking Back Control - Control | AIM: To increase the perception of control  Objectives: Understanding burnout and its consequences on decision making, Reframing situations to feel more in control. Using recovery techniques as a way to prevent burnout and feel more in control. | I can decide when to take a break  I have a say in my own work speed  I have a choice in deciding how to do at work  I have a choice in deciding what I do at work  I have some say over the way I work  My working time can be flexible | JDR model^1^; basic psychological needs (Autonomy)^2^; Conservation of Resources Model^5^; Recovery^6^; Psychological Detachment^7^; CBT (Circle of Control, and the link between thoughts, feelings and emotions)^8^; self-compassion^9^. |
| Shaping social support - Support | AIM: To increase social connections and social support.  Objectives; To understand the health implications of a lack of social support. To reconnect via small daily interactions, and build on existing social connections linked to self-identify. | If work gets difficult  my colleagues will help me  I get help and support I need from colleagues  I receive the respect at work I deserve from my colleagues  My colleagues are willing to listen to my work-related problem | Basic psychological needs (Connectedness)^2^; CBT (Transactional Analysis)^10^; Positive Psychology (acts of kindness, gratitude)^11^; Social Psychology^12^; JDR - Social resources^13^. |

1. Bakker, A. B., & Demerouti, E. (2007). The job demands-resources model: State of the art. Journal of managerial psychology,22(3), 309-328.
2. Ryan, R. M., & Deci, E. L. (2017). Self-determination theory: Basic psychological needs in motivation, development, and wellness. Guilford Publications.
3. Tice, D. M., & Baumeister, R. F. (2018). Longitudinal study of procrastination, performance, stress, and health: The costs and benefits of dawdling. In Self-Regulation and Self-Control(pp. 299-309). Routledge.
4. Tse, D. C., Nakamura, J., & Csikszentmihalyi, M. (2019). Beyond challenge-seeking and skill-building: Toward the lifespan developmental perspective on flow theory. The Journal of Positive Psychology, 1-12.
5. Hobfoll, S. E., & Shirom, A. (2001). Conservation of resources theory: Applications to stress and management in the workplace.
6. Bennett, A. A., Bakker, A. B., & Field, J. G. (2018). Recovery from work‐related effort: A meta‐analysis. Journal of Organizational Behavior, 39(3), 262-275.
7. De Bloom, J., Geurts, S. A., Taris, T. W., Sonnentag, S., de Weerth, C., & Kompier, M. A. (2010). Effects of vacation from work on health and well-being: Lots of fun, quickly gone. Work & Stress, 24(2), 196-216.
8. Larsson, A., Hooper, N., Osborne, L. A., Bennett, P., & McHugh, L. (2016). Using brief cognitive restructuring and cognitive defusion techniques to cope with negative thoughts. Behavior Modification,40(3), 452-482.
9. Leary, M. R., Tate, E. B., Adams, C. E., Batts Allen, A., & Hancock, J. (2007). Self-compassion and reactions to unpleasant self-relevant events: the implications of treating oneself kindly. Journal of Personality and Social Psychology, 92(5), 887.
10. Marlow, S. L., Lacerenza, C. N., Paoletti, J., Burke, C. S., & Salas, E. (2018). Does team communication represent a one-size-fits-all approach?: A meta-analysis of team communication and performance. Organizational Behavior and Human Decision Processes, 144, 145-170.
11. Curry, O. S., Rowland, L. A., Van Lissa, C. J., Zlotowitz, S., McAlaney, J., & Whitehouse, H. (2018). Happy to help? A systematic review and meta-analysis of the effects of performing acts of kindness on the well-being of the actor. Journal of Experimental Social Psychology, 76, 320-329.
12. Haslam, C., Jetten, J., Cruwys, T., Dingle, G., & Haslam, S. A. (2018). The New Psychology of Health. Unlocking the Social Cure, 201.
13. DeLongis, A., Folkman, S., & Lazarus, R. S. (1988). The impact of daily stress on health and mood: psychological and social resources as mediators. Journal of Personality and Social Psychology,54(3), 486.
