# Supplementary material for: Efficacy of the Digital Therapeutic Mobile App BioBase to Reduce Stress and Improve Mental Well-Being Among University Students: Randomized Controlled Trial
Source: JMIR Mhealth Uhealth. 2020 Apr 6;8(4):e17767. doi: 10.2196/17767 (PMC7171562; doi:10.2196/17767)
Supplement: Multimedia Appendix 2 [file mhealth_v8i4e17767_app2.docx]

Multimedia Appendix 2. Summary of the Linear Mixed Model on DASS-21 Depression scores  over the duration of the intervention (T0, T1 and T2) in the intervention and wait-list control groups.

|  | **DASS 21 Depression** | | |
| --- | --- | --- | --- |
| *Predictors* | *Estimates* | *CI* | *p* |
| (Intercept) | 20.72 | 15.22 – 26.21 | **<0.001** |
| Group | -2.14 | -5.57 – 1.30 | 0.224 |
| T 1 | -4.83 | -9.09 – -0.57 | **0.027** |
| T 2 | -7.68 | -12.03 – -3.32 | **0.001** |
| Group:TimeT1 | 1.30 | -1.37 – 3.97 | 0.340 |
| Group:TimeT2 | 1.97 | -0.75 – 4.68 | 0.157 |
| **Random Effects** | | | |
| σ^2^ | 3.27 | | |
| τ_00_ _Time:ID_ | 24.98 | | |
| τ_00_ _ID_ | 66.10 | | |
| ICC | 0.97 | | |
| N _Time_ | 3 | | |
| N _ID_ | 123 | | |
| Observations | 368 | | |
| Marginal R^2^ / Conditional R^2^ | 0.042 / 0.967 | | |
