# Supplementary material for: Efficacy of the Digital Therapeutic Mobile App BioBase to Reduce Stress and Improve Mental Well-Being Among University Students: Randomized Controlled Trial
Source: JMIR Mhealth Uhealth. 2020 Apr 6;8(4):e17767. doi: 10.2196/17767 (PMC7171562; doi:10.2196/17767)
Supplement: Multimedia Appendix 3 [file mhealth_v8i4e17767_app3.docx]

Multimedia Appendix 3. App Engagement


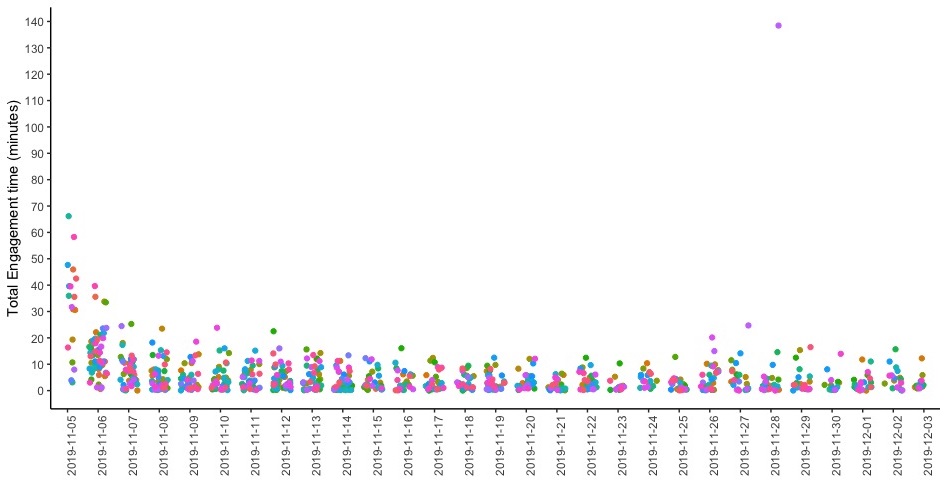


Figure 1. Total engagement time per day per user for the whole duration of the intervention (29 days). Dots = individual users.


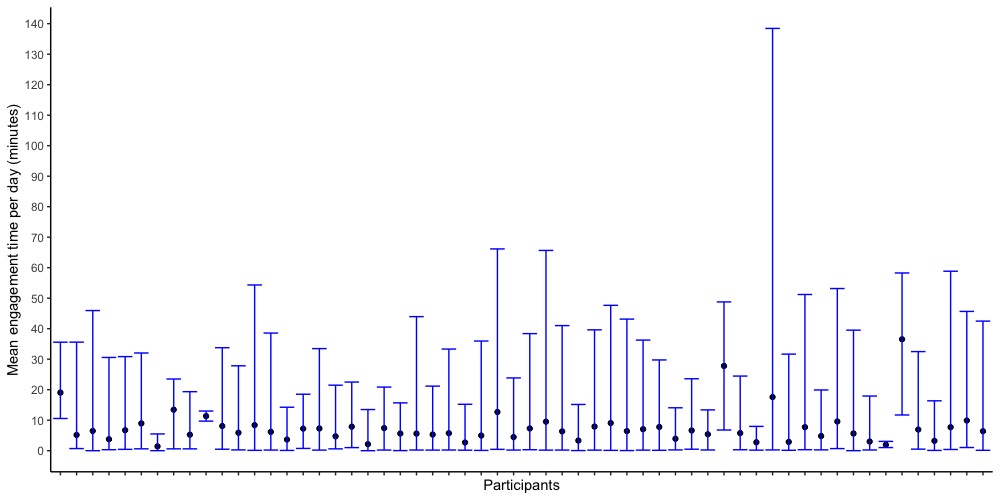


Figure 2. Average engagement time per day per user. Dots = individual users; Error bars: min/max engagement time over the duration of the intervention.
