# Supplementary material for: Efficacy of the Digital Therapeutic Mobile App BioBase to Reduce Stress and Improve Mental Well-Being Among University Students: Randomized Controlled Trial
Source: JMIR Mhealth Uhealth. 2020 Apr 6;8(4):e17767. doi: 10.2196/17767 (PMC7171562; doi:10.2196/17767)
Supplement: Multimedia Appendix 4 [file mhealth_v8i4e17767_app4.docx]

Multimedia Appendix 4**.** Engagement time and differences in anxiety and wellbeing


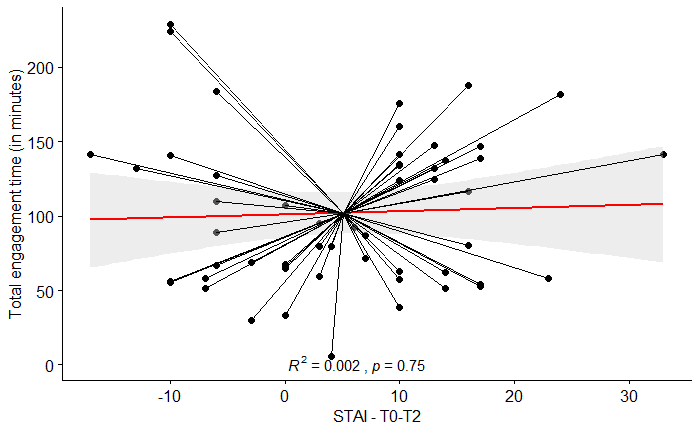


Figure 3. Correlation between total engagement time per user for the whole duration of the intervention (29 days) and differences in STAI-S-6 scores from baseline to T2 (4-weeks).


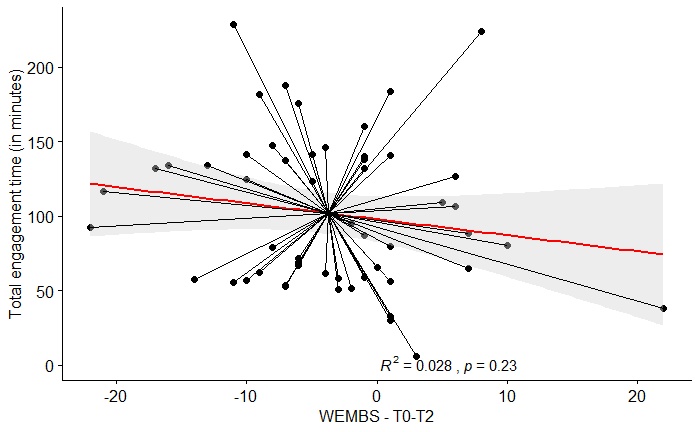


Figure 4. Correlation between total engagement time per user for the whole duration of the intervention (29 days) and differences in WEWMBS scores from baseline to T2 (4-weeks).
